# New Insights into the Tumor Microenvironment Utilizing Protein Array Technology

**DOI:** 10.3390/ijms19020559

**Published:** 2018-02-13

**Authors:** Wei Huang, Shuhong Luo, Rob Burgess, Yu-Hua Yi, Gordon F. Huang, Ruo-Pan Huang

**Affiliations:** 1 RayBiotech, Inc., Guangzhou, 79 Ruihe Road, Huangpu District, Guangzhou 510600, China; w_huang@Raybiotech.cn (W.H.); sluo@raybiotech.com (S.L.); sophia@raybiotech.cn (Y.-H.Y.); 2 South China Biochip Research Center, 79 Ruihe Road, Huangpu District, Guangzhou 510600, China; 3 RayBiotech, Inc., 3607 Parkway Lane, Norcross, GA 30092, USA; rob@raybiotech.com (R.B.); gordon.huang@raybiotech.com (G.F.H.)

**Keywords:** protein array, antibody array, microarray, tumor microenvironment, cancer stem cell, cancer

## Abstract

The tumor microenvironment (TME) is a considerably heterogeneous niche, which is created by tumor cells, the surrounding tumor stroma, blood vessels, infiltrating immune cells, and a variety of associated stromal cells. Intercellular communication within this niche is driven by soluble proteins synthesized by local tumor and stromal cells and include chemokines, growth factors, interferons, interleukins, and angiogenic factors. The interaction of tumor cells with their microenvironment is essential for tumorigenesis, tumor progression, growth, and metastasis, and resistance to drug therapy. Protein arrays enable the parallel detection of hundreds of proteins in a small amount of biological sample. Recent data have demonstrated that the application of protein arrays may yield valuable information regarding the structure and functional mechanisms of the TME. In this review, we will discuss protein array technologies and their applications in TME analysis to discern pathways involved in promoting the tumorigenic phenotype.

## 1. Introduction

Cancer has been long viewed as a heterogeneous disease involving aberrant mutations in cancer cells. Cancer cells acquire cell autonomous hyperproliferative and limitless survival capacities [1]. Emerging evidence indicates that the tumor microenvironment (TME) plays an important role in tumor progression and treatment response. The TME consists of proliferating tumor cells, infiltrating inflammatory cells, the extracellular matrix, blood vessels, and a variety of associated stromal cells and soluble proteins [2]. Interaction between tumor cells and the TME influences tumor initiation, progression, and ultimately patient prognosis [3]. These changes are not a consequence of a single protein’s function but rather involve multiple proteins that function in many pathways and networks. Intercellular communication is driven by a complex and dynamic network of soluble proteins such as cytokines, chemokines, growth factors, and matrix-degrading enzymes that enhance tumor cell proliferation and invasion and inhibit tumor cell apoptosis. Although enzyme-linked immunosorbent assay (ELISA) is the most common method used to measure protein expression levels, this method is limited to the detection and quantification of only one protein. Protein arrays enable the parallel detection of hundreds of proteins in minimal sample volumes. By using this approach, the expression levels of hundreds to thousands of proteins can be detected and even fully quantified. This review focuses on the application of protein arrays in assessing the TME.

## 2. The Tumor Microenvironment

**Figure 1:**
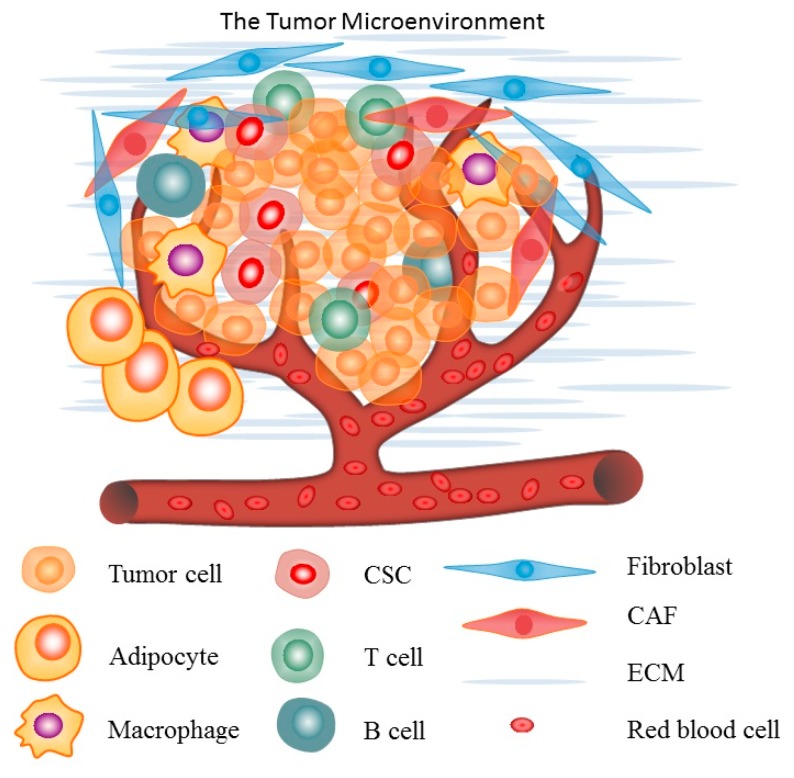
An illustration of the tumor microenvironment.

Cancer is a systemic disease. Apart from malignant cells, the TME contains cells of the immune system, vasculature and lymphatics, fibroblasts, pericytes, and adipocytes (Figure 1) [4]. Interactions between malignant and non-transformed cells create the TME and accumulating evidence suggests that the TME plays an important role in tumorigenesis and tumor progression. Thus, the pathogenesis of cancer is largely dependent upon the interaction of the tumor with the other components of the TME [5]. The first evidence that non-cancerous tissue elements might affect tumor formation and growth came from the field of inflammation. The link between chronic inflammation and cancer was first proposed by Rudolf Virchow in 1863 when he observed that inflammatory cells frequently infiltrate tumor stroma [6]. Recently, increasing evidence supports a role for endothelial cells, macrophages, and cancer-associated fibroblasts in promoting tumor growth and progression. Adaptive and innate immune cells represent a significant component of the TME, and it has been proposed that both innate and adaptive immunity play important roles in immune-surveillance and tumor destruction [7,8].

## 3. Cytokines and Cell Networks in the Tumor Microenvironment

### 3.1. Cytokines

Cytokines are secreted proteins that mediate cell-to-cell communication. These proteins include chemokines, growth factors, interferons, interleukins, and angiogenic factors, and they have been shown to play critical roles in many diseases [9]. In solid tumors, tumor and stromal cells synthesize these proteins, resulting in the development of a complex and dynamic network [4,10]. There is increasing evidence that these soluble proteins can modulate cancer progression, response to chemotherapy, cancer immunity, and metastatic status. Thus, not only do cytokines provide defense against cancer cells but they also promote cancer cell growth at all stages of cancer development in the TME [11]. Many cytokines are pleiotropic and redundant, meaning that a single cytokine may induce a wide range of effects in various cell and tissue types and multiple cytokines may have similar effects on a single cell type. For example, Ozaki et al. described the sharing of the γc receptor by the interleukins (IL)-2, IL-4, IL-7, IL-9, IL-15 and IL-21; the sharing of IL-2Rβ by IL-2 and IL-15; the sharing of IL-4Rα and IL-13Rα1 by IL-4 and IL-13 in the TME [12,13]. Cytokines often function as a complex network of tightly controlled signaling pathways. Their individual and even global signals are modulated by each other through cytokine-specific receptors present on the surface of virtually all cell types [9]. Accurate quantification of cytokine levels in biological samples is conventionally accomplished using ELISA. Although ELISA is popular and has been widely used for a number of years, it is limited to measuring only one cytokine per experiment, and is a considerably low-throughput, time-consuming, and expensive assay to use to quantify a large panel of proteins. Conversely, protein arrays are able to detect and even fully quantify multiple proteins simultaneously, thus saving precious sample and time and are a good economic alternative to ELISA-based analyses.

### 3.2. Cell Networks in the Tumor Microenvironment (TME)

Apart from malignant cells, the TME contains significant immune and stromal cell populations, which play distinct roles in the modulation of the local tumor environment, either promoting or inhibiting tumorigenesis. These populations include innate and adaptive immune cells such as T lymphocytes, dendritic cells (DC), B cells, macrophages, polymorphonuclear leukocytes, and, rarely, natural killer (NK) cells, all of which constitute essential components of the TME [2].

Macrophages, often referred to as tumor-associated macrophages (TAMs) when present in the TME, are either derived from peripheral reservoirs such as the bone marrow (BM) and spleen or reside in the original stromal environment [14]. TAMs are abundant in most human cancers and are associated with a poor prognosis. TAMs directly affect tumorigenesis, cancer cell growth, neo-angiogenesis, and extracellular matrix remodeling [15]. Macrophages are functionally plastic and can alter their polarization state to accommodate different physiological conditions. Macrophage polarization can range from M1 (classically activated) to M2 (alternatively activated), and said polarization affects their functionality in the TME. M1 macrophages produce pro-inflammatory cytokines, participate in antigen presentation, and play an anti-tumorigenic role [16]. In contrast, M2 macrophages produce different cytokines, promote anti-inflammatory responses, and have a pro-tumorigenic role [3,17].

Fibroblasts synthesize and deposit the extracellular matrix (ECM) through production and secretion of ECM components [18]. Cancer-associated fibroblasts (CAFs) are stromal cells found in cancerous tissues which share similarities with myofibroblasts (MFs) [19]. CAFs are one of the most crucial components of the TME, playing a vital role in supporting and promoting tumor growth and progression [20]. It is becoming clear that the crosstalk between the cancer cells and the CAFs induces cancer progression as well as resistance to cancer therapies through secretion of proteins, exosomes, and ECM remodeling factors [21].

In addition, many tumors contain phenotypically and functionally heterogeneous cancer cells and are maintained by a small subpopulation of cells that display stem cell properties [22]. These cancer stem cells (CSCs) are characterized by their capacity for self-renewal, differentiation, and tumor initiation. CSC functionality is often regulated by the TME and there is increasing evidence that CSCs mediate metastasis and contribute to treatment resistance [23,24]. Yet, the interaction between CSCs and their microenvironment may also provide novel therapeutic targets for treatment.

## 4. Protein Array Technology

**Figure 2:**
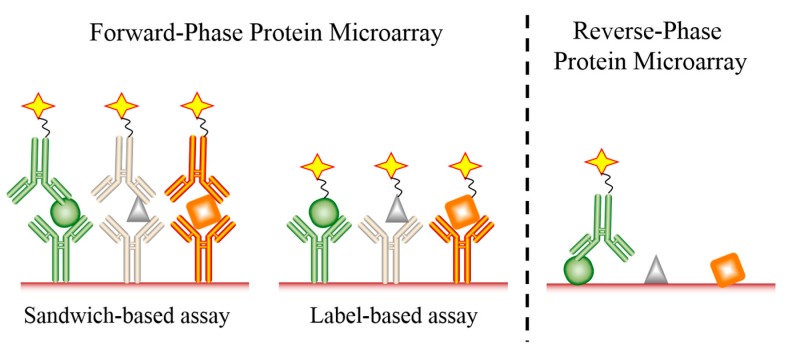
An illustration of both forward and reverse-phase protein microarrays. Proteins are identified as circles, triangles and squares. Detection fluorophores or enzymes are identified as stars.

Over the last several decades, DNA microarray technologies have been developed and successfully used for analyzing the whole transcriptome [25]. However, gene expression profiling through mRNA characterization often does not directly correlate with protein levels [26]. Moreover, it is the level of proteins which reflects the real-time physiological and pathological state of an organ, tissue, or cells far more accurately than RNA levels. In addition, post-translational modifications, which cannot be detected at the mRNA level, may play an important role in disease development and progression. Protein microarrays allow for the simultaneous and rapid analysis of thousands of proteins and their corresponding modifications in biological samples such as serum, plasma, and tissue or cell lysate. Protein microarrays have thus become well established tools in basic and applied biological research, including TME research. Two platforms of protein arrays have been developed: the forward-phase protein microarray (FPPM) and the reverse-phase protein microarray (RPPM) (Figure 2) [27]. The FPPM is the most frequently used format, consisting of an array of well-defined, immobilized capture molecules that allow the simultaneous analysis of a large number of different parameters in a biological sample [26]. The two main types of FPPMs are label-based assays and sandwich-based assays. These methods are complementary and have both advantages and disadvantages.

One type of FPPM which has been widely used to detect proteins in biological samples is the antibody-based sandwich microarray. In this format, the microarray is based upon antibody pairs that recognize different epitopes of the same protein. One antibody is immobilized on the solid-phase support to capture the protein, and the other serves as a detection antibody that is often coupled to biotin. In this way, quantifiable levels of the target protein bound to the array can be measured using streptavidin labeled with a fluorophore, enzyme, or some other detection molecule [28]. Some advantages of this method include an increased sensitivity, specificity, and dynamic range of the assay. Moreover, sandwich-based microarrays can also be used for quantitative analysis. However, a major disadvantage of this method is the limited number of proteins that can be analyzed simultaneously due to antibody cross-reactivity issues, with the potential cross-reactivity among detection antibodies increasing with additional analytes. In addition, antibody-based sandwich assays are often difficult to develop due to the limited availability of matched pairs of antibodies, and this may be compounded by the possibility that purified antigens may not be available for each target [11,29].

A second type of FPPM is the label-based assay, which utilizes one antibody printed on a solid-phase support to capture the target protein. In label-based microarrays, all proteins in a sample are directly labeled with a tag, such as biotin or a fluorescent dye, which allows for detection after antibody capture. Since no detection antibody is required, the possibility of antibody cross-reactivity is eliminated in this system, making the development of extremely high-density microarrays possible [30]. In fact, hundreds or even thousands of target proteins can be detected simultaneously using this single antibody capture and detection approach. Another advantage of label-based assays is the ability to incubate two different samples, each labeled with a different tag, on the same array. Thus, multi-sample simultaneous analysis can be accomplished, saving resources and time. The main disadvantage of single antibody label-based assays is inefficient labeling of all proteins in the sample, which may limit detection as well as sensitivity and specificity [11,29].

Serving as a complementary approach to measure protein expression are RPPMs. In contrast to the FPPM, the RPPM is based upon the immobilization of proteins present in biological samples onto a solid-phase support. The array is then probed with a highly specific antibody to simultaneously determine the relative abundance of the target proteins in the sample. The signals can be measured by fluorescent, chemiluminescent, or colorimetric detection methods, and can also be amplified by coupling the detection antibody with tyramide-based biotin signal amplification systems [31,32]. This technique allows for a variety of biological samples to be easily and efficiently characterized, such as blood samples, cultured supernatants, and cell or tissue lysates. RPPMs have been successfully used in the study of many diseases [26,33].

## 5. Protein Arrays Reveal Unique Insight into the Tumor Microenvironment

Antibody microarrays are one of the high-throughput protein analysis techniques capable of detecting hundreds of proteins simultaneously. Multiple lines of evidence have shown that antibody microarrays have proven to be reliable research tools in analysis of the TME. The following is a discussion of several examples that illustrate the utility of antibody microarrays in TME research (summarized in Table 1).

### 5.1. TME Regulation of Tumor Progression and Metastasis

Cancer is not an autonomous disease but rather depends upon numerous factors for survival, progression, and metastasis [34,35]. Interactions between cancer cells and the TME help to drive the tumorigenic phenotype, and accumulating evidence suggests that a complex and dynamic network of soluble proteins—such as cytokines, chemokines, growth factors, and inflammatory and matrix remodeling enzymes—play pivotal roles in the bidirectional communication between cancer cells and the TME [4]. Therefore, it is critical to understand the molecular mechanisms of this process. Antibody microarrays enable the parallel detection of hundreds of proteins, and have been widely used in the investigation of molecular mechanisms of tumor progression and metastasis. For example, the transcription factor interferon regulatory factor-8 (IRF-8) is crucial for myeloid cell development and immune response and also acts as a tumor suppressor gene. To investigate the role of IRF-8 in the cross-talk between melanoma cells and tumor-infiltrating leukocytes, Mattei et al. [36] transplanted B16-F10 melanoma cells into IRF-8-deficient (IRF-8^−/−^) mice and investigated melanoma cell growth rates in this in vivo system. They noted that the melanoma cells grew significantly faster and had enhanced lung metastasis and reduced DC and T cell infiltration compared to transplants in wild-type (WT) mice. In addition, the researchers performed co-culture experiments by co-culturing splenocytes from melanoma-bearing IRF-8^−/−^ or WT mice with B16-F10 melanoma cells, and found that IRF-8 expression in B16 cells was significantly upregulated by splenocytes from tumor-bearing WT mice compared with the control mice. To identify the key factors responsible for IRF-8 up-regulation, the authors employed antibody microarrays designed to detect various cytokines and analyzed supernatants from splenocyte/B16 cell co-cultures. The results showed that IL-3, IL-6, and IL-10 were differentially expressed in splenocytes from tumor bearing WT mice compared with tumor-bearing IRF-8^−/−^, naïve WT and naïve IRF-8^−/−^ mice. Taken together, these results suggest that IRF-8 regulates melanoma progression and invasiveness by soluble factors released by immune cells in the TME [36].

Anderberg et al. [37] found that transgenic expression of the growth factor PDGF-CC in a mouse model of tumorigenesis accelerated tumor growth through recruitment of CAFs into tumors. To identify the molecular mechanisms involved in fibroblast recruitment, the authors implemented antibody microarrays to measure 96 different secreted growth factors, cytokines, chemokines, and transmembrane receptors in pooled lysates from B16/PDGF-CC tumors compared with B16/mock tumors. The results showed that the growth factors fibroblast growth factor (FGF)-2 and osteopontin were significantly higher in B16/PDGF-CC tumor lysates compared with controls. The results were further confirmed by immunoprecipitation and Western blot characterizing samples from three independent B16/PDGF-CC and B16/mock tumors. Subsequent studies found that osteopontin is predominantly expressed by CAFs in B16/PDGF-C tumors. These studies thus identified key regulators of local fibroblast recruitment and suggest that PDGF-CC enhances the growth of B16 tumors by recruitment of CAFs that secrete osteopontin [37].

Alkasalias et al. [38] studied the inhibition of tumor cell proliferation caused by local environmental effects, specifically focusing on whirly fibroblast inhibition of PC3 monomeric red fluorescent protein (mRFP) tumor cell growth. The authors found that both live and formaldehyde-fixed confluent monolayers of whirly fibroblasts can inhibit the proliferation of PC3 mRFP tumor cells. They then sought to discern the molecular players involved in this inhibition and noted that conditioned media from confronted cultures (CCM) significantly increased the inhibitory capacity of fixed whirly fibroblasts compared with fixed fibroblasts treated with either non-confronted conditioned media (NCM) or medium alone. In contrast, NCM showed no effect on the inhibitory capacity of fixed fibroblasts compared with medium alone. Plus, CCM inhibited the motility of tumor cells cultured on a fixed fibroblast monolayer, whereas NCM had no effect on the motility of PC3 mRFP cells on a fixed monolayer of fibroblasts. To identify proteins or factors secreted by fibroblasts that inhibit both tumor cell proliferation and motility, the authors measured the expression profiles of 507 proteins in conditioned media from non-confronted BJhTERT, confronted BJhTERT + PC3 cells, and PC3 cells alone using a biotin label-based antibody microarray. The results revealed the differential expression of nine proteins, eight of which (GDF-15, DKK1, EDA-A2, EMAP-II, Galectin-3, CXCL2, Nidogen1 and uPA) were significantly increased in the conditioned media from the confrontation sample compared with controls. MMP3 was the only protein that showed down-regulation upon confrontation compared with non-confrontation samples. Taken together, the results of this study identify several key soluble regulators of fibroblast-based tumor cell growth inhibition [38].

Triple-negative breast cancer (TNBC) patients have a higher risk of both local and distal cancer recurrence and metastases. TNBC has been postulated to result from the lack of the estrogen, progesterone, and Herceptin (HER2/neu) receptor expression. TNBC is also characterized by reduced expression of metastasis suppressors, such as RAF kinase inhibitory protein (RKIP), that inhibit tumor invasiveness. Frankenberger et al. [39] found that CCL5 overexpression in RKIP^+^ tumors restored recruitment of pro-metastatic TAMs and intravasation. To define the potential mechanism of this recruitment, the authors employed a cytokine antibody microarray to measure protein expression levels in TAM-conditioned media from both RKIP + CCL5 tumors and RKIP tumors. The array data showed that several cytokines, including VEGF-A, VEGF-D, OPN, LGALS3, SLPI, MMP-12, sTNFR2, and PGRN were significantly increased in TAM-conditioned media isolated from RKIP + CCL5 tumors compared to controls. Further studies found that either sTNFR2 or PGRN significantly induced tumor cell invasion. These results revealed the identity of factors that regulated metastasis and suggest that pro-metastatic factors counter-regulated by RKIP and CCL5 directly promote human breast cancer cell invasion [39].

Song et al. [40] found that hepatic stellate cells (HSCs) activated by low pH promoted hepatocellular carcinoma (HCC) metastasis in vitro and in vivo. Using antibody arrays, the authors found that osteopontin secretion from HSCs was increased in an acidic environment and was the driving force behind the migration of HCC cells. Furthermore, osteopontin expression levels were shown to be directly associated with myofibroblasts, and both α-smooth muscle actin (SMA) and osteopontin levels were powerful predictors of poor prognosis in HCC patients. Therefore, HSCs activated by acidic TMEs represent a novel mechanism for HCC metastasis, and provides a potential therapeutic strategy for HCC intervention [40].

### 5.2. Identification of Potential Drug Targets

Drug discovery is a complex and expensive process that usually begins with a search for novel drug targets [47]. Proteins are most often the targets of choice as they can act as receptors, modulators, and regulators of the disease phenotype. Most drugs act by binding to specific proteins, thereby changing their biochemical and/or biophysical activities, with multiple consequences for various functions [48]. As it relates to cancer, interactions between malignant and non-transformed cells occur by a complex network of soluble proteins in the TME, and these proteins may act as targets for therapeutic intervention. Antibody microarray technology may allow for the rapid and comprehensive dissection of this network; thus, increasing the odds for the identification of valuable drug targets, thereby greatly facilitating the search for new drugs. Several examples are provided below of drug targets discovered by utilizing antibody microarray technology.

Multiple studies have reported that macrophages are associated with both tumor metastasis and related poor survival in cancer patients. Fu et al. [41] co-cultured HCC cells with phorbol myristic acetate (PMA)-treated Tamm-Horsfall Protein 1 (THP-1) macrophages. The results showed that macrophages promoted HCC cell migration and invasion and induced HCC cells. To identify the mechanism by which macrophages promote HCC invasion, the authors employed an antibody microarray to analyze a panel of cytokines expressed in HCC/macrophage co-culture and HCC control culture supernatants. The results revealed that MIP-3α, TNF-α, RANTES, MCP-1, IL-6, IL-8, IL-1β, and GRO-α were significantly increased in both co-cultured MHCC-97H and Hep-G2 cells compared with controls. Further analyses with ELISA directly correlated with the microarray findings and confirmed that the expression levels of TNF-α, IL-6, IL-8, and IL-1β in the media from co-cultured HCC cells were significantly increased compared with controls. The expression level of IL-8 was nearly 100-fold higher in supernatants of HCC cells cultured with macrophages relative to HCC control cells. A series of follow-up experiments demonstrated that IL-8 induces epithelial-to-mesenchymal transition (EMT) and promotes HCC cell migration and invasion by activating the JAK2/STAT3/Snail pathway. By employing protein microarray technology, this study may have yielded novel therapeutic targets for developing new HCC therapies [41].

To investigate whether the interaction between cancer cells and TAMs promotes tumor metastasis, Su et al. [15] cultured freshly isolated human monocytes in conditioned medium from either mesenchymal-like breast cancer cells or epithelial-like breast cancer cells. Using this conditioned medium analysis experimental protocol, they demonstrated that media from mesenchymal-like cancer cells activated macrophages to transition into a TAM-like phenotype, suggesting that mesenchymal-like cancer cells secrete soluble factor(s) that activate macrophage conversion. In order to identify key soluble factors driving this macrophage activation, the authors employed an antibody microarray to analyze conditioned media from MCF-7 cells, MCF-7 cells that underwent EMT, and MDA-MB-231 cells. The data showed that GM-CSF, IL-8, CCL2, GRO, and GROα were significantly increased in MCF-7 cells that have undergone EMT and MDA-MB-231 cells compared with MCF-7 cells. Further experiments demonstrated that treatment with GM-CSF alone was sufficient and necessary for mesenchymal-like cancer cells to induce macrophage activation to a TAM-like phenotype. The activated macrophages subsequently produced CCL18, which induces EMT in cancer cells, thereby forming a positive feedback autoregulatory loop. Subsequent studies by Su et al. demonstrated that the positive feedback loop between GM-CSF and CCL18 is essential to promote the metastasis of breast cancer cells and is associated with poor prognosis in breast cancer patients. These findings strongly suggest that GM-CSF may be a novel therapeutic target for the inhibition of metastasis [15]. Taken together, these studies support the use of antibody microarrays in the investigation of new potential drug targets for a variety of disease states.

### 5.3. Drug Resistance

Drug resistance is a fundamental problem that limits the effectiveness of chemotherapies in the treatment of cancer patients. Drug resistance is often attributed to functional gene mutations, amplifications, or epigenetic changes that influence the uptake, metabolism or export of drugs from single cells. However, a number of studies suggest that mechanisms involving the TME also mediate resistance to drug therapy and that this may occur by both direct contact between cells in the TME as well as through the presence of local soluble secreted factors. This is evidenced by the studies of Straussman et al. [42], who discovered that the TME can directly induce innate resistance to therapy. In an elegant study, the authors employed an in vitro co-culture system to analyze cancer cell–stromal cell–drug interactions. Forty-five human cancer cell lines were cultured either alone or in combination with 23 human stromal cell lines in the presence of 35 anti-cancer drugs. The results showed that stromal cells confer innate resistance to cancer cells. The authors further explored the mechanism of stroma-mediated innate resistance to the RAF inhibitor PLX4720. They co-cultured 7 BRAFV600E melanoma cell lines with 18 stromal cell lines and subsequently treated each culture with an anti-cancer RAF inhibitor. They found that, out of the 23 stromal cell lines, six conferred innate resistance by cancer cells. To further investigate the mechanism of stroma-mediated innate resistance to the RAF inhibitor, the authors employed an antibody microarray to measure the expression profiles of 567 secreted proteins in the conditioned media from six rescuing stromal cell lines and 12 non-rescuing stromal cell lines. The data revealed that hepatocyte growth factor (HGF) was best correlated with PLX4720 resistance. Subsequent studies found that HGF resulted in reactivation of the microtuble associated protein kinase (MAPK) and phosphotidyl inositol 3 kinease (PI3K/AKT) pathways, which conferred resistance to the RAF inhibitor [42].

Previous studies have demonstrated that CAFs can promote the growth and invasion of cancer cells in the TME. Recent studies strongly suggest that CAFs also promote therapeutic resistance, mainly through the secretion of multiple cytokines. Sun et al. [44] applied a cytokine antibody microarray to detect the secreted soluble factors derived from conditioned medium of non-cancer-associated fibroblasts (NAF), fibroadenoma fibroblasts (FADs), paracarcinoma fibroblasts (PCFs), and CAFs with four breast cancer molecular phenotypes (luminal A, luminal B, HER2^+^ and TNBC). The array data showed that the levels of IL-6, IL-8, and GRO (CXCL1, CXCL2 and CXCL3) were significantly increased in the CAF-CM compared with the levels in NAF, FAD, and PCF-CM. To determine which of these factors may promote tamoxifen resistance, they tested the ability of recombinant human IL-6, IL-8, and CXCL3 to promote cancer cell survival in the presence of tamoxifen. The results demonstrated that only IL-6 induced tamoxifen resistance, whereas IL-8 or CXCL3 had no significant effect. To demonstrate this effect further, they decreased IL-6 expression using short hairpin RNA (shRNA) in CAF18 cells. Knockdown of IL-6 expression significantly enhanced the drug sensitivity of these cells. These experiments suggest that secretion of IL-6 by CAFs promotes luminal breast cancer tamoxifen resistance [43].

### 5.4. Cancer Stem Cells and the Tumor Microenvironment

Many tumors are maintained by a small internal subpopulation of cells that display stem cell properties which mediate tumor progression and metastasis [22]. These CSCs are regulated by complex interactions with the TME through networks of proteins [47,49]. Recent evidence has shown that the TME provides essential cues for both the maintenance of CSCs and the promotion of cancer stem cell self-seeding at metastatic sites.

Cancer stem cells have been shown to play important roles in tumor progression and tumor recurrence following therapy in many types of tumors, including HCC. Several studies have showed that the presence of TAMs in HCC patients is associated with a poor prognosis, yet it is not clear whether TAMs interact with CSCs during HCC development. Wan et al. [44] found that HepG2 cells or Hep3B cells co-cultured with TAMs can promote expansion of CD44 + HCC cells, and increase cancer sphere-forming capacity. To investigate how TAMs induced HCC CSC expansion, the authors analyzed the expression profiles of 82 proteins in supernatants from HepG2/TAM co-cultures, HepG2 or TAMs alone using a cytokine antibody microarray. The results indicated that the expression of IL-6 was significantly increased in HepG2/TAM co-cultures compared with HepG2 or TAMs supernatants, and these results were confirmed by ELISA. Furthermore, by adding recombinant human IL-6 to HepG2 or Hep3B cell cultures, the authors observed an increase in CD44^+^ cells and sphere formation. In addition, IL-6 expression levels directly correlated with stages of human HCC and the detection of CSC markers. These results identified IL-6 as a key regulator of cancer stem cell growth and suggests that TAM-secreted IL-6 promotes CSC expansion in HCC [44].

The TME contains many different stromal cells including one primary type: BM-derived myeloid cells (BMFs). Numerous studies have demonstrated that BMFs enhance tumor development and invasion, specifically in gastric cancer [50,51]. For example, Zhu et al. [45] found that BMFs enhanced tumorigenesis and tumor growth when co-injected with gastric cancer cells in vivo. Additional studies by Zhu and colleagues found that co-cultured BMFs or BMF-conditioned medium (BMF-CM) induced sphere formation of gastric cancer cells, which expressed stem cell properties and exhibited features of self-renewal, epithelial-to-mesenchymal transition and tumor initiation. To identify possible BMF-derived factors contributing to the induction of the CSC phenotype, the authors applied antibody microarrays in an analysis of conditioned media from BMF-CM and co-culture-CM of BMFs and MKN28 cells. The array data showed that murine IL-6 levels were significantly higher in co-culture-CM than that in BMF-CM. These studies revealed that the soluble factors BMF-derived IL-6/HGF and cancer cell-derived TGF-β1 are the primary mediators of interactions between BMFs and gastric cancer cells and that these factors drive cancer cell stemness and promote tumorigenesis [45].

Myofibroblasts (MFs) have long been understood to drive stemness in CSCs, although the mechanisms, until recently, have remained unknown. The Wnt signaling cascade has also been speculated to be involved in promoting a stem-like phenotype in cancer cells. To directly investigate whether MFs could affect Wnt signaling in CSCs, Vermeulen et al. [46] co-cultured a colonic MF cell line with CSCs. In a parallel experiment, CSCs were cultured with conditioned medium derived from the same MF cell line. These studies demonstrated a downregulation in classical markers for differentiation in CSCs exposed to either MFs or corresponding conditioned media. To identify which, if any, cytokine factors may be involved in this process, the authors employed a cytokine antibody microarray to screen 79 secreted proteins in the conditioned media from MF and control cell lines. The array data showed that hepatocyte growth factor (HGF) was significantly upregulated in MFs compared to controls. Furthermore, HGF was also expressed at higher levels in MFs derived from primary colon cancer. Further studies revealed that HGF secreted by MFs modulated nuclear β-catenin activity through the receptor c-Met and thereby affected CSC features in colorectal tumor cells [46].

## 6. Conclusions

Protein microarray analysis is a cutting-edge technique that enables the high-throughput, comprehensive, parallel detection and quantification of hundreds of proteins. Many different platforms of protein microarrays have been developed and are commercially available, including FPPM and RPPM. Among FPPM technologies, sandwich-based antibody arrays and label-based antibody arrays are the two main types. The RPPM format is ideal for cell lysate analysis when high quality target-specific antibodies are available. Both methods can perform successful analyses of various sample types such as serum, plasma, urine, other bodily fluids, cell culture supernatants, tissue or cell lysates, and resected tumor specimens. Label-based antibody arrays use one antibody to capture the target protein and detection occurs via labeling of all proteins in a sample, while sandwich-based antibody arrays require a pair of antibodies to capture and detect the target protein. Therefore, sandwich-based antibody arrays have higher specificity, sensitivity, and reproducibility compared with label-based antibody arrays. However, sandwich-based antibody arrays require high-affinity and specific antibodies for the discrete detection of target proteins. Antibody microarrays may be applied to many different fields of research, including the study of various types of cancer, systemic autoimmune disorders, neurodegenerative diseases, and cardiovascular diseases. In addition, the TME plays an important role in tumorigenesis and tumor progression, and interactions between tumor cells and other components of the local microenvironment depend upon soluble proteins. Protein microarrays allow for the simultaneous and rapid analysis of hundreds of proteins in samples and may represent ideal tools for the rapid identification and characterization of critical factors present in the TME.

## Figures and Tables

**Table 1:** Summary of protein arrays used in analysis of the tumor microenvironment.

Cell Type	Experimental Method	Array Type	Array Result	Citation
B16-F10 cells	in vitro cell co-culture	sandwich-based FPPM	IL-3, IL-6 and IL-10 are released by immune cells in the TME	[36]
PDGF-C transfected B16-F10 cell-derived tumors	in vivo mouse tumor model	sandwich-based FPPM	FGF-2 and osteopontin expression was significantly higher in B16/PDGF-C tumor lysates compared with controls	[37]
BJhTERTs and PC3 mRFP tumor cells	in vitro cell culture	label-based FPPM	GDF-15, DKK1, EDA-A2, EMAP-II, Galectin-3, CXCL2, Nidogen1 and uPA were significantly increased and MMP3 significantly decreased in CM from the confrontation sample compared with controls	[38]
RKIP + BM1 tumor-derived TAMs	in vitro cell culture	sandwich-based FPPM	VEGF-A, VEGF-D, OPN, LGALS3, SLPI, MMP-12, sTNFR2 and PGRN were significantly increased in TAM-CM isolated from RKIP+CCL5 tumors compared with controls	[39]
LX-2 cells	in vitro cell culture	sandwich-based FPPM	osteopontin secretion was increased in an acidic environment and was the driving force behind the migration of HCC cells	[40]
MHCC-97H, Hep-G2, and THP-1 cells	in vitro cell co-culture	sandwich-based FPPM	MIP-3α, TNF-α, RANTES, MCP-1, IL-6, IL-8, IL-1β and GRO-α were significantly increased in both co-cultured MHCC-97H and Hep-G2 cells compared with controls	[41]
MCF-7, EMT-MCF-7, and MDA-MB-231 cells	in vitro cell culture	sandwich-based FPPM	GM-CSF, IL-8, CCL2, GRO and GROα were significantly increased in MCF-7 cells that have undergone EMT and MDA-MB-231 cells compared with MCF-7 cells	[15]
18 stromal cell lines	in vitro cell co-culture	sandwich-based and label-based FPPMs	HGF was best correlated with PLX4720 resistance	[42]
NAFs, FADs, PCFs and CAFs	in vitro cell co-culture	sandwich-based FPPM	IL-6, IL-8 and GRO (CXCL1, CXCL2 and CXCL3) levels were consistently higher in the CAF-CM than in the NAF, FAD and PCF-CM	[43]
HepG2, Hep3B, and TAMs	in vitro cell co-culture	sandwich-based FPPM	IL-6 was significantly increased in HepG2/TAM co-cultures compared with HepG2 or TAMs cultures	[44]
BMFs and MKN28 cells	in vitro cell co-culture	sandwich-based FPPM	IL-6 levels were significantly higher in co-culture-CM than those in BMF-CM	[45]
myofibroblasts and CSCs	in vitro cell culture	sandwich-based FPPM	HGF was significantly upregulated in MFs compared to controls.	[46]

Abbreviations: FPPM: Forward Phase Protein Microarray; IL-3: Interleukin 3; FGF-2: Fibroblast Growth Factor 2; DKK1: Dickkopf-related protein 1; EDA-A2: Ectodysplasin A; EMAP-II: Endothelial-Monocyte Activating Polypeptide II; CXCL2: Chemokine (C-X-C motif) ligand 2; uPA: Urokinase-type-plasminogen-activator; MMP3: Matrix Metalloproteinase 3; CM: Conditioned Media; RKIP: RAF kinase inhibitor protein; BMF: Bone marrow-derived myofibroblasts; VEGF-A: Vascular Endothelial Growth Factor Type A; VEGF-D: Vascular Endothelial Growth Factor Type D; OPN: Osteopontin; LGALS3: Lectin, Galactoside-Binding, Soluble Protein 3; SLPI: Secretory Leukocyte Protease Inhibitor; sTNFR2: Soluble Tumor Necrosis Factor Receptor Type 2; MIP-3α: Macrophage Inflammatory Protein-3 α; TNF-α: Tumor Necrosis Factor α; RANTES: Regulated on Activation, Normal T Cell Expressed and Secreted; MCP-1: Monocyte Chemoattractant Protein Type 1; GM-CSF: Granulocyte Macrophae Colony Stimulating Factor; CCL2: Chemokine (C-C motif) Ligand Type 2; GRO: Growth Regulated Protein; FAD: Flavin Adenine Dinucleotide; HGF: Hepatocyte Growth Factor; MFs; Myofibroblasts.
